# Type II Collagen-Specific B Cells Induce Immune Tolerance in Th1-Skewed, Th2-Skewed, and Arthritis-Prone Strains of Mice

**DOI:** 10.3390/cells10040870

**Published:** 2021-04-12

**Authors:** Shukkur M. Farooq, Hossam M. Ashour

**Affiliations:** 1 Department of Pharmacy Practice, Eugene Applebaum College of Pharmacy and Health Sciences, Wayne State University, Detroit, MI 48201, USA; mdfarooqs76@gmail.com; 2 Department of Integrative Biology, College of Arts and Sciences, University of South Florida, St. Petersburg, FL 33701, USA; 3 Department of Microbiology and Immunology, Faculty of Pharmacy, Cairo University, Cairo 11562, Egypt

**Keywords:** ACAID, peripheral tolerance, immune privilege, regulatory T cells, collagen type II, B cells

## Abstract

Antigen-specific regulatory T cells play key immune suppressive roles in autoimmune disease models and regulate the peripheral tolerance achieved via anterior chamber-associated immune deviation (ACAID). Articular cartilage has type II collagen (CII), which is a potent autoantigen protein in arthritis. There has not been much research on the clinical importance of CII-associated diseases. Moreover, the capability of CII to induce immune tolerance has not been previously assessed. We reported that delivery of CII either directly into the eye or via intravenous injection of CII-specific ACAID antigen presenting cells (APCs) can induce ACAID. Here, we hypothesized that peripheral tolerance can be induced following adoptive transfer of *in vitro* generated CII-specific ACAID B cells to naive mice. Delayed hypersensitivity (DTH) assays were used to assess the suppressive ability of adoptively transferred B cells. Immune responses of ACAID B cell-injected mice were significantly suppressed following challenges with CII as compared to positive controls. This effect was replicated in three different strains of mice (C57BL/6, BALB/c, and DBA/1). Thus, CII-specific ACAID B cells were able to induce immune tolerance in Th1-skewed, Th2-skewed, and arthritis-prone mice. ACAID B cell-mediated tolerance induced by CII could have therapeutic implications for the treatment of CII-mediated autoimmune diseases.

## 1. Introduction

A potent antigen-specific peripheral immune tolerance mechanism termed anterior chamber-associated immune deviation (ACAID) can be induced by introducing antigens into the anterior chamber (AC) of the eye [1,2,3]. ACAID leads to the prevention of inflammation and related deleterious peripheral immune responses [1,2,3]. Antigen-specific regulatory T cells (Tregs) help maintain immune tolerance through ACAID [1,2]. ACAID can be induced by different antigens [4]. The main hallmarks of ACAID are antigen-specific regulation of systemic Th1 immune responses and impaired antigen-specific delayed-type hypersensitivity (DTH) responses [5,6]. Antigens entering the AC are processed by resident ocular tissue F4/80^+^ antigen-presenting cells (APCs) which internalize the antigens and gain access into the bloodstream, thymus, and eventually the spleen [7,8]. These ocular APCs interact with marginal zone B cells, γδ T cells, and NK T cells in the spleen and induce the differentiation of CD8^+^ efferent Tregs and CD4^+^ afferent Tregs [7]. Generation of CD4^+^ CD25^+^ Tregs and CD8^+^ Tregs by the ocular APCs involves the development of antigen-specific B cells in the spleen [9]. Moreover, tolerance developed by *in vitro* or *in vivo* generated antigen-specific ACAID B cells has been reported by us and others [7,10,11]. B cells have been shown to play critical tolerogenic roles in ACAID by presenting an-tigens through Major Histocompatibility Complex (MHC) I and MHC II [7]. ACAID-mediated peripheral tolerance can potentially regulate autoimmunity through the suppression of Th1 and Th2 effector mechanisms in an antigen-specific manner [3,12].

In rheumatoid arthritis (RA), type II collagen (CII), the main constituent of articular cartilage, is a possible autoantigen [13]. Indeed, collagen-induced arthritis (CIA), an RA-like disease, can be induced in rodents via immunization using CII plus adjuvant [14]. Autoantibodies towards both native and citrullinated CII have been identified in the serum and synovial fluid of RA patients [15,16]. The present investigation was meant to address two main questions: (1) whether the adoptive transfer of CII-specific ACAID B cells could induce ACAID in the recipient mice; (2) whether there are differences in responses among three different strains of recipient mice (BALB/c, C57BL/6, and DBA/1). The chosen mice strains represent a good mix of diversified immune responses and susceptibility to arthritis. More specifically, BALB/c mice are Th2-skewed, C57BL/6 mice are Th1-skewed and arthritis-prone, and DBA/1 mice are arthritis-prone.

## 2. Materials and Methods

### 2.1. Mice

C57BL/6, BALB/c, and DBA/1 mice (6–8 weeks of age) were obtained from Jackson Laboratories (Bar Harbor, ME, USA). All animals were housed and maintained in the animal care facility of the Eugene Applebaum College of Pharmacy and Health Sciences, Wayne State University.

### 2.2. Generation of ACAID APCs 

Generation of ACAID APCs *in vitro* from C57BL/6, BALB/c, or DBA/1 mice was done as described earlier [7]. Briefly, APCs were cultured overnight (2 × 10^6^ cells/mL) in complete RPMI 1640 with 10 mg/mL CII and 2–5 ng/mL TGF-β2 (R&D Systems, Minneapolis, MN, USA) at concentrations similar to those present in the aqueous humor of the eye.

### 2.3. Immunization by Subcutaneous Injection

Subcutaneous injection of 250 µg of CII (Sigma-Aldrich, St. Louis, MO, USA) emulsified 1:1 in complete Freund’s adjuvant (CFA; Sigma-Aldrich, St. Louis, MO, USA) was performed, and 200 µL of the CII/CFA emulsion was injected into each animal.

### 2.4. Generation of ACAID B Cells

We used an *in vitro* culture system that was previously used to generate ACAID APCs and ACAID B cells capable of inducing the generation of Tregs that express the same phenotype as those induced by the AC injection of the antigen [7,17]. In accordance with the procedure, CII-pulsed ocular-like APCs were generated *in vitro* and were cocultured for 48 h with B cells isolated from the spleens of normal C57BL/6, BALB/c, or DBA/1 mice using CD45R (B220) microbeads (Miltenyi Biotec, Bergisch Gladbach, Germany). Residual macrophages were killed by treating the non-adherent B cell population with anti-F4/80^+^plus complement as done previously [7,18]. Next, C57BL/6, BALB/c, or DBA/1 mice were injected intravenously with these ACAID-inducing B cells (2–4 × 10^6^ cells/mouse). Trypan blue exclusion assay was used to measure the viability of B cells immediately before adoptive transfer (>95% viability).

### 2.5. DTH Assay

Inhibition of DTH responses at the efferent arm of the immune response has long been used as a measure for ACAID induction [5,19]. DTH assays were performed in a similar manner as previously described [20]. Briefly, naive recipient mice were adoptively transferred with CII-specific ACAID B cells that were generated *in vitro*. Recipient mice were immunized subcutaneously with CII+CFA after 7 days. On day 14, mice were challenged in the ears with CII, and a DTH assay was conducted after 24 h and 48 h. Positive control mice received subcutaneous immunization with CII but not ACAID B cells. Negative control mice were neither injected with ACAID B cells nor CII-immunized via the subcutaneous route. The left ear pinna was injected with 500 μg CII in 20 μL, and the right ear pinna was injected with 20 µL of 10 mM acetic acid (internal control). A Mitutoyo engineer micrometer was used to measure ear swelling before and 24 h after CII injection. The results were measured as follows: Specific ear swelling = (24 h measurement–0 h measurement) for left ear − (24 h measurement–0 h measurement) for right ear. Results after 48 h were calculated in a similar manner: Specific ear swelling = (48 h measurement–0 h measurement) for left ear − (48 h measurement–0 h measurement) for right ear. Each group had five animals. The same protocol was used for all three strains.

### 2.6. Statistics

Student’s t test was used to assess statistical significance. Data was expressed as mean ± SD for all experimental measurements.

## 3. Results

### 3.1. CII-Specific ACAID B Cells Induced Peripheral Tolerance

*In vitro*-generated or *in vivo*-generated ACAID B cells can be adoptively transferred to naive recipients for the induction of ACAID [21]. DTH assays were used to test whether *in vitro*-generated CII-specific ACAID B cells can induce peripheral tolerance in recipient mice. The regulatory effects of CII-specific ACAID B cells were tested by measuring the extent of prevention of the DTH ear-swelling responses induced by immune cells in the recipient mice. We followed the same experimental protocol for a previous report in which we showed that ~1 × 10^6^ ACAID B cells were required for the generation of OVA-specific ACAID [10]. 

**Figure 1:**
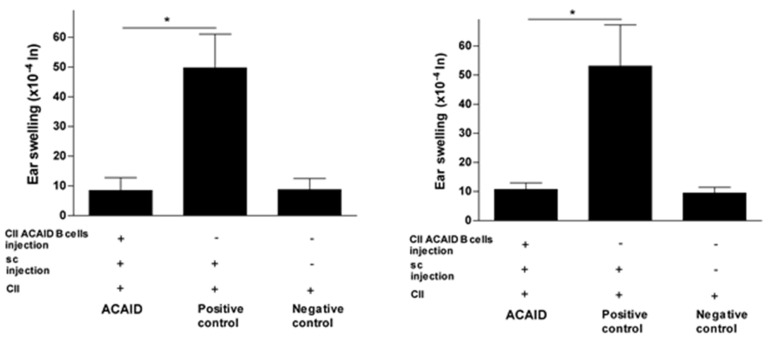
*In vitro* generated type II collagen (CII)-specific anterior chamber-associated immune deviation (ACAID) B cells inhibited CII-induced delayed hypersensitivity (DTH) responses in BALB/c mice. The *in vitro* generation of CII-specific ACAID B cells and the DTH assay were described in the Materials and Methods section. CII-specific ACAID B cells were intravenously injected to induce ACAID followed by subcutaneous immunization with CII/CFA on day 7. On day 14, mice were challenged with CII (500 μg in 20 μL) intradermally in the left ear pinna, and 20 μL of acetic acid alone was injected into the right ear pinna as an internal control. ACAID induction was confirmed by suppression of ear swelling responses after 24 h (left panel) and 48 h (right panel). The positive control mice were subcutaneously immunized with CII/CFA on day 7 and with CII on day 14, whereas the negative control mice only received intradermal injection of CII on day 14. * *p* < 0.05 were considered to be statistically significant.

**Figure 2:**
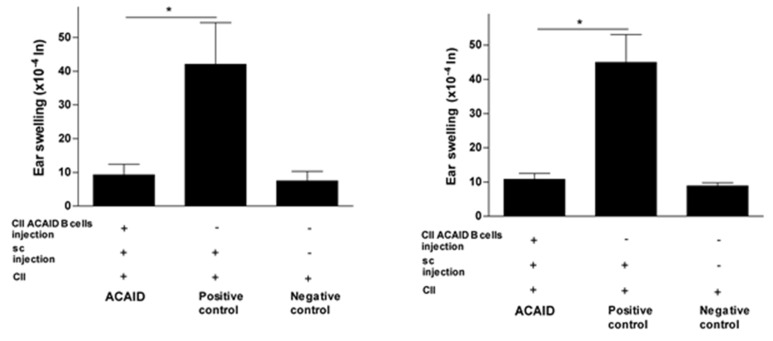
*In vitro* generated CII-specific ACAID B cells inhibited CII-induced DTH responses in C57BL/6 mice. The *in vitro* generation of CII-specific ACAID B cells and the DTH assay were described in the Materials and Methods section. CII-specific ACAID B cells were intravenously injected to induce ACAID followed by subcutaneous immunization with CII/CFA on day 7. On day 14, mice were challenged with CII (500 μg in 20 μL) intradermally in the left ear pinna, and 20 μL of acetic acid alone was injected into the right ear pinna as an internal control. ACAID induction was confirmed by suppression of ear swelling responses after 24 h (left panel) and 48 h (right panel). The positive control mice were subcutaneously immunized with CII/CFA on day 7 and with CII on day 14, whereas the negative control mice only received intradermal injection of CII on day 14. * *p* < 0.05 were considered to be statistically significant.

**Figure 3:**
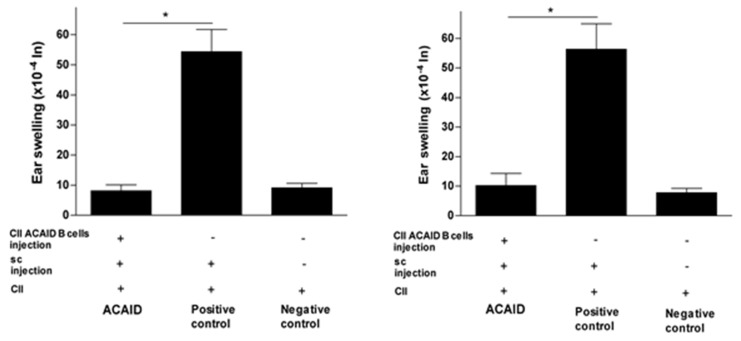
*In vitro* generated CII-specific ACAID B cells inhibited CII-induced DTH responses in DBA/1 mice. The *in vitro* generation of CII-specific ACAID B cells and the DTH assay were described in the Materials and Methods section. CII-specific ACAID B cells were intravenously injected to induce ACAID followed by subcutaneous immunization with CII/CFA on day 7. On day 14, mice were challenged with CII (500 μg in 20 μL) intradermally in the left ear pinna, and 20 μL of acetic acid alone was injected into the right ear pinna as an internal control. ACAID induction was confirmed by suppression of ear-swelling responses 24 h (left panel) and 48 h (right panel). The positive control mice were subcutaneously immunized with CII/CFA on day 7 and with CII on day 14, whereas the negative control mice only received intradermal injection of CII on day 14. * *p* < 0.05 were considered to be statistically significant.

The results in Figure 1, Figure 2 and Figure 3 revealed that mice receiving the adoptive transfer of ACAID B cells developed significantly lower DTH responses than positive control mice, but these responses were not significantly different from DTH responses in negative control mice. Thus, the adoptively transferred B cells managed to suppress DTH responses in recipient mice (Figure 1, Figure 2 and Figure 3).

### 3.2. CII-Specific B Cells Induced Tolerance in C57BL/6 Mice, BALB/c Mice, and DBA/1 Mice

The CII-specific regulatory effect was consistent after 24 h and 48 h of the intradermal ear injection with CII and was also consistent across all three mice strains used in the study (Figure 1, Figure 2 and Figure 3). The statistical significance of DTH suppression in the ACAID B cell group as compared to the positive control group was as follows: BALB/c mice: * *p* = 0.0032 (24 h) and * *p* = 0.0021 (48 h); C57BL/6 mice: * *p* = 0.0009 (24 h) and * *p* = 0.0014 (48 h); DBA/1 mice: * *p* = 0.00008 (24 h) and * *p* = 0.0002 (48 h).

This is the first study to show the induction of CII-specific peripheral tolerance using CII-specific ACAID B cells. Furthermore, these cells were shown to have the ability to successfully induce ACAID in the Th1-skewed C57BL/6 mice, the Th2-skewed BALB/c mice, and the arthritis-prone DBA/1 mice.

## 4. Discussion

Tolerance can be defined as a phenomenon in which suppression of the immune reaction due to presence of one or more mechanisms results in a state of unresponsiveness to a specific antigen [22,23]. Thus, tolerance is not just the mere absence of an immune response but is an active regulatory process. Understanding the induction of peripheral immune T cell tolerance can help us devise new therapeutic strategies to regulate the balance between tolerance and immunity in cases such as autoimmune diseases, tumor immunity, and transplant tolerance. 

Immune privilege has been described in the eye [24,25], brain [26,27,28], testis [29,30], and hair follicle [31,32]. The entry of soluble antigens into immune privileged sites such as the AC of the eye is an important process that triggers the activation of immune tolerance, which protects from inflammation [33]. Communication between the AC of the eye and peripheral immune cells occurs through the process of ACAID, which results in the generation of systemic antigen-specific tolerance [7,34]. In ACAID, T cells can be tolerized by professional B cells resulting in the suppression of immune responses [7]. This B-cell-triggered tolerization of the T cell compartment is caused by different antigen presentation mechanisms [7].

Tolerance of the CD8^+^ T cell compartment can be induced by B cells directly or indirectly. Direct recognition of antigen presented on B cells by CD8^+^ T cells through MHC class I results in the development of tolerance. This may cause deletion or anergy of the T cell compartment and can also lead to the development of CD8^+^ T suppressor cells (active suppression). The generation of both CD4^+^ and CD8^+^ Tregs involves critical roles of B cells from the spleen [7,17,21]. Using both *in vitro* and *in vivo* ACAID models, it was obvious that ACAID B cells must express both MHC class I and II molecules for the generation of Tregs [7]. Thus, immune tolerance induction via the eye may require that the antigen-presenting B cells utilize both MHC class I and MHC II molecules for antigen presentation to the Tregs. It would be beneficial to conduct detailed cellular and molecular studies that directly assess the possible expansion of CD8^+^ Tregs as a result of their interaction with the antigen-presenting B cells in ACAID.

There are several forms of T cell tolerance induced by B cells [35]. Delivering antigens intravenously, orally, or via the AC of the eye can induce T cell tolerance [21,35,36]. The antigen-presenting role for B cells could be a universal mechanism for inducing T cell tolerance in models that rely on the presence of B cells. The present study is the first to report that CII-specific ACAID B cells have the ability to induce peripheral tolerance in BALB/c, C57BL/6, and DBA/1 mice. In order to develop a successful therapeutic treatment strategy for autoimmune diseases such as rheumatoid arthritis, this CII-specific ACAID B cell-mediated approach could be useful.

## Data Availability

All relevant data is contained in the manuscript.
